# Active Site Conformational Dynamics in Human Uridine Phosphorylase 1

**DOI:** 10.1371/journal.pone.0012741

**Published:** 2010-09-14

**Authors:** Tarmo P. Roosild, Samantha Castronovo

**Affiliations:** Department of Drug Development, Nevada Cancer Institute, Las Vegas, Nevada, United States of America; University Paris Diderot-Paris 7, France

## Abstract

Uridine phosphorylase (UPP) is a central enzyme in the pyrimidine salvage pathway, catalyzing the reversible phosphorolysis of uridine to uracil and ribose-1-phosphate. Human UPP activity has been a focus of cancer research due to its role in activating fluoropyrimidine nucleoside chemotherapeutic agents such as 5-fluorouracil (5-FU) and capecitabine. Additionally, specific molecular inhibitors of this enzyme have been found to raise endogenous uridine concentrations, which can produce a cytoprotective effect on normal tissues exposed to these drugs. Here we report the structure of hUPP1 bound to 5-FU at 2.3 Å resolution. Analysis of this structure reveals new insights as to the conformational motions the enzyme undergoes in the course of substrate binding and catalysis. The dimeric enzyme is capable of a large hinge motion between its two domains, facilitating ligand exchange and explaining observed cooperativity between the two active sites in binding phosphate-bearing substrates. Further, a loop toward the back end of the uracil binding pocket is shown to flexibly adjust to the varying chemistry of different compounds through an “induced-fit” association mechanism that was not observed in earlier hUPP1 structures. The details surrounding these dynamic aspects of hUPP1 structure and function provide unexplored avenues to develop novel inhibitors of this protein with improved specificity and increased affinity. Given the recent emergence of new roles for uridine as a neuron protective compound in ischemia and degenerative diseases, such as Alzheimer's and Parkinson's, inhibitors of hUPP1 with greater efficacy, which are able to boost cellular uridine levels without adverse side-effects, may have a wide range of therapeutic applications.

## Introduction

Uridine phosphorylase (UPP; EC 2.4.2.3) is a ubiquitous enzyme that catalyzes the reversible phosphorolysis of uridine and analogous compounds to uracil and ribose-1-phosphate, playing an important role in pyrimidine salvage and regulation of uridine homeostasis [1]–[3]. Most mammals, including humans, possess two isoforms of the enzyme, UPP1 [4] and UPP2 [5], of which UPP1 has been much more extensively studied. Interest in understanding the activity of human uridine phosphorylase (hUPP) stems from its role in the activation of pyrimidine nucleoside analogues used in chemotherapy, such as 5-fluorouracil (5-FU) [6] and its prodrug, capecitabine. In this case, the enzyme converts 5-FU to 5-fluorouridine, which is subsequently further activated by uridine kinase to create 5-fluorouridine monophosphate. Multiple further downstream metabolites of 5-FU exert anti-cancer activity through disruption of RNA synthesis, misincorporation into DNA, or inhibition of thymidylate synthase, the activity of which is essential for DNA synthesis and repair. Other research has shown that some tumours have increased levels of hUPP activity, a finding that may partly explain the tissue selectivity of these chemotherapeutic agents [7], [8]. More recent investigations have explored using hUPP inhibitors to boost cellular uridine concentrations, as a means of limiting the toxic effects of fluoropyrimidine nucleoside exposure to healthy tissues during the course of treatment [9], [10]. Compounds such as 5-benzylacyclouridine (BAU) [11] have been tested for their ability to increase the maximum tolerated dosage and therapeutic index of 5-FU through this uridine-mediated cyto-protective phenomenon [12].

A fundamental understanding of the underlying structural mechanisms behind the catalytic activity of this enzyme has been established through extensive structural analysis of bacterial UPPs, starting with *E. coli* UPP (EcUPP) [13]–[16] and then the closely-related *S. typhimurium* homologue [17]–[19]. More recently, multiple structures of the human enzyme, hUPP1 [20], its bovine homologue, bUPP1 [21], and a UPP from the parasitic protozoa, *Trypanosoma brucei* [22], have been determined. These structures have revealed unexpected differences in variations of this enzyme. Most interestingly, the hexameric, trimer-of-dimers organization of prokaryotic UPPs has been dissociated in favour of strictly dimeric complexes in eukaryotic organisms. These studies are also uncovering unique differences in the molecular details of the architecture of these enzymes that may be critical to discovering novel compounds with increased efficacy in modulating this enzyme's activity for the development of better therapeutic regimens.

Here we present the crystallographic structure of hUPP1 bound to 5-FU. This structure reveals previously unknown conformational flexibility in loops proximate to this enzyme's active sites that impact the structure-guided design of new inhibitors of this protein. These insights regarding the structural dynamics of hUPP1 will be useful both for improving our understanding of this enzyme's role in the activation of fluoropyrimidines and in identifying strategies for more effectively modulating this protein's activity through medications.

## Results

### hUPP1 structure when bound to 5-fluorouracil

**Figure 1:**
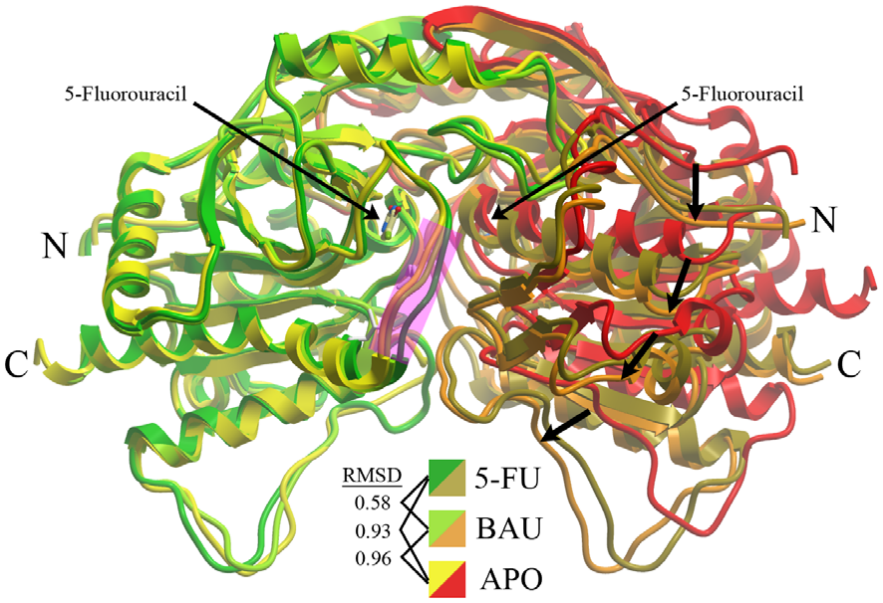
Structural comparison of hUPP1 with varying ligands. Overlay of the structures of hUPP1 bound to 5-FU, BAU, or ligand-free (APO) reveals the high degree of retention of the global fold of the enzyme when binding either substrate or inhibitor. The position of the two 5-FU molecules within the symmetric active sites at the dimer interface is also shown. In this illustration, the green/yellow monomers are least-squares aligned (R.M.S.D.s shown in angstroms) and the resulting displacement of the backbone traces of the partnering chains (arrows) reveals the interdomain flexibility of hUPP1. Between aligned monomers binding either 5-FU or BAU, there is a noticeable structural difference only in the conformation of a loop proximate to the active site (magenta).

One of the unexpected findings of the first structures of hUPP1 was the discovery that the domains of the enzymatic dimer are flexibly linked, allowing an interdomain motion from an “open” ligand-free conformation to a “closed” catalytically-active structure [20]. The structure of hUPP1 bound to 5-FU provides further insight into this motion, having been crystallized in a transitional, intermediate position roughly 80% of the way from “open” to “closed” (Figure 1). The observed rotational movement hinges around a stable pivot point on one face of the enzyme that contains an extensive interdomain interface formed by overlapping strand-turn-strand elements that are not present in prokaryotic homologues [20]. The constraints imposed by the hexameric ring structure of bacterial UPPs likely prevent the occurrence of a similar phenomenon in these enzymes and it has not been observed in any of the structures of these proteins. This interdomain motion may have interesting consequences on the kinetics and regulation of dimeric eukaryotic enzymes relative to their better-characterized prokaryotic counterparts. The observed structural flexiblity links the two active sites and predicts that substrates that stablize the ‘closed’ conformation of the enzyme by associating with residues from both protein chains will cooperatively increase the affinity of other substrates that also bind across the dimer interface. Indeed, cooperative binding of phosphate and ribose-1-phosphate has recently been confirmed through extensive *in vitro* analysis of the biochemistry of recombinant hUPP1 [23].

It is notable, that the interdomain motion between folds within a hUPP1 dimer is accompanied by nearly imperceptible changes in the conformational structure of the individual domains. The overall R.M.S.D. of main chain atoms from ligand-free to BAU-bound for aligned monomers is less than 1.00 Å. The differences are even less comparing BAU-bound and 5-FU-bound enzymes, with structural differences limited almost exclusively to a loop lining the back side of the active site pocket (Figure 1, magenta highlight).

### Coordination of 5-FU within the hUPP1 active site

**Figure 2:**
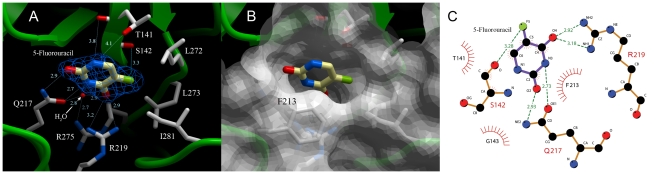
5-Fluorouracil binding to hUPP1. (**A**) 5-FU is coordinated by residues restricted to the individual monomers of the hUPP1 dimer, in contrast to the binding of BAU that traverses the dimer interface. As expected, Gln217 and Arg219, the key uridine-discriminating residues, form multiple hydrogen bonds with one face of the uracil base. This face also includes a well-coordinated, buried water molecule that associates with 5-FU and creates stabilizing bonds with both Gln217 and Arg275. Additional favourable interactions may be formed by both the backbone carbonyl and side chain hydroxyl groups of Thr141, although the geometry observed in the crystal structure is not consistent with hydrogen bonding. The fluorine moiety resides in a hydrophobic pocket created by Leu272, Leu273 and Ile281, and forms a hydrogen bond with Ser142. Electron density from a 2F_o_-F_c_ map contoured at 1.5σ is shown for the ligand (blue wire). (**B**) Surface representation from the same perspective emphasizes the depth and fit of the active site for the pyrimidine substrate. The position of Phe213, which was omitted from (A) for clarity, is also illustrated. This residue caps the active site and forms hydrophobic, herringbone stacking interactions with the uracil ring. (**C**) Schematic map of the contacts between hUPP1 and 5-FU as analyzed by LigPlot [37].

Analysis of electron density distribution at the enzyme's active site reveals density in omit maps consistent with bound 5-FU (Figure 2). The coordination of 5-FU by the protein is exactly as seen previously for *E. coli* UPP with 5-FU [15], *S. typhimurium* UPP with 5-FU [18], and bovine UPP1 with 5-FU [21]. The binding of uracil is stabilized by a network of hydrogen-bonds created by Gln217, Arg219, Arg275 and a single deeply buried water molecule. All of these elements are strictly conserved among known UPPs and have been proposed to form a UPP-specificity motif for distinguishing those enzymes with uridine preference from among the larger family of nucleoside phosphorylases [22]. The fluorine moiety of 5-FU forms a hydrogen bond with Ser142 and is otherwise closely encased in a cluster of hydrophobic residues including Leu272, Leu273 and Ile281. These latter residues, which are key to binding the benzyl modification of high affinity inhibitors such as BAU, are also the only distinguishing active site residues when comparing eukaryotic and prokaryotic enzymes (equivalent *E. coli* residues are Ile 220, Val221 and P229). This is an important consideration when contemplating generating selectivity in such competitive inhibitors between the two enzymes, as would be needed for the development of effective antibiotics targeting only bacterial homologues of this protein [19].

### Substrate-induced conformational changes

**Figure 3:**
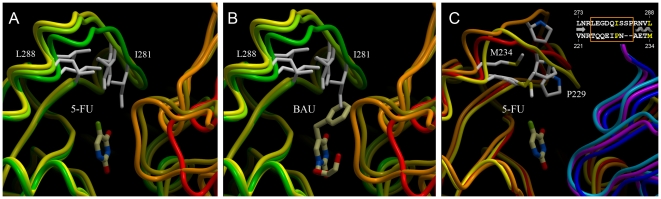
Conformational dynamics of a hUPP1 active site loop. (**A**) Comparison of the structure of the loop lining the back of the hUPP1 active site when bound to 5-FU (green), BAU (lime), or ligand-free (yellow), reveals that this region is somewhat mobile and able to close around substrate upon its binding. (**B**) Overlay of the BAU molecule with the known structures of hUPP1 shows that the benzyl moiety of this inhibitor displaces Ile281 from its normal substrate binding position to accommodate the extra bulkiness of this molecule. It is notable how similar the BAU-bound and ligand-free conformations of hUPP1 are, suggesting that BAU fits the naturally occurring structure of the protein in the absence of substrate. (**C**) While the new structure of hUPP1 reveals some degree of flexibility in the back-side active site loop, the conformational range of this region of hUPP1 is substantially less than that of the equivalent part of *E. coli* UPP, which closes more tightly when bound to 5-FU (yellow) and opens wider in the absence of ligand (orange) when compared with its BAU-inhibited structure (red). The increased rigidity of the human enzyme is likely due to the insertion of two additional residues into this loop region, including a proline (inset).

Comparison of the architectures of BAU-bound and 5-FU bound monomers of hUPP1 reveals that substantial structural changes are restricted to a single loop region toward the back side of the active site. This loop has been demonstrated in *E. coli* UPP to undergo an ‘induced-fit’ restructuring from a flexible, disordered form to an active-site capping position upon ligand binding [15]. In contrast, earlier structures of hUPP1 had revealed no conformational differences between BAU-bound and ligand-free states for this loop and comparably low thermal factors in both structures for this region of the protein, leading to the suggestion that this loop might be rigid in the human enzyme [20]. The 5-FU-bound structure shows that this conclusion is inaccurate, as the loop clearly closes around the small fluorine moiety of 5-FU (Figure 3A). While this flap-like structure is clearly less mobile in the human enzyme than in its prokaryotic equivalent, its retained ability to adjust its shape to accommodate various altered chemical forms of uracil/uridine has important implications for understanding how to strategically exploit this dynamic element in the design of better enzyme inhibitors. It is certainly noteworthy that BAU binds hUPP1 with this loop in nearly an identical conformation as found in the ligand-free structure, suggesting that this compound binds to a naturally occurring, low-energy state of the enzyme (Figure 3B). As this region includes the three active site-distinguishing hydrophobic residues mentioned earlier, this additional feature further impacts the strategy for developing selective inhibition of only one homologue over others (Figure 3C).

**Figure 4:**
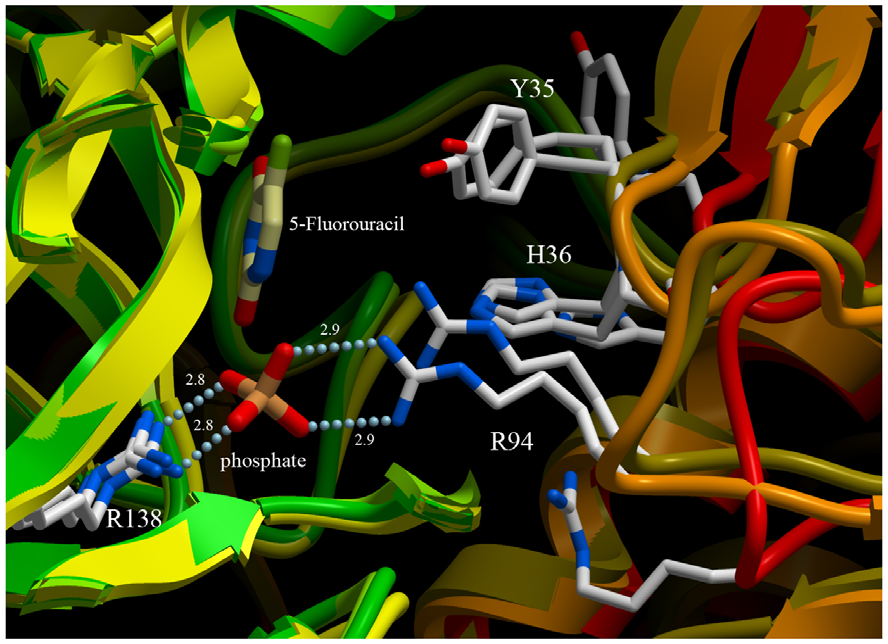
Inter-domain flexibility of hUPP1. Illustration highlights conformational changes at the dimer interface proximate to the active site, overlaying the 5-FU-bound structure (gold), the BAU-bound structure (orange), and ligand-free structure (red). Despite a lack of molecular contacts between residues from the partnering subunit and the 5-FU ligand, the critical residues for binding natural substrates adopt conformations close to those seen in the BAU-bound structure, where they are stabilized by the formation of favourable molecular interactions, and not the conformations revealed in the ligand-free structure. The location of the phosphate ion from the BAU-bound structure is shown for orientation, but not found to be occupied in the 5-FU-bound structure.

As the interdomain hinge is in a transitional, intermediate state in the 5-FU-bound structure of hUPP1, it was expected that there would also be substantial conformational changes in the active site residues that reach from one domain to influence ligand binding in the partnering domain's active site. Earlier studies of hUPP1 suggested that hinge closure was driven by the creation of energetically favourable contacts between residues from the one chain with the small molecules bound to the other chain's active site [20]. Specifically, in the BAU-bound structure of hUPP1, three partner-subunit residues assisted in the coordination, respectively, of the benzyl moiety (Tyr35), the ribose group (His36), and phosphate ion (Arg94). These chemical groups are all absent from the 5-FU-bound structure, eliminating every interdomain protein-ligand interaction. In spite of the lack of these stabilizing associations, all of these residues adopt conformations similar to those observed in the BAU-bound structure (Figure 4). This suggests that the active, ligand-binding conformations of these residues are low energy, naturally-adopted rotamers, thus favouring substrate association and catalysis of phosphorolysis. It is also interesting that while both the BAU-bound and 5-FU structures were crystallized under very similar conditions, only the former was found to have bound phosphate, presumably chelated from trace contamination in the purification or crystallization solutions. This is likely a result of the benzyl and sugar groups of the BAU inhibitor stabilizing a fully “closed” enzyme configuration in which the two phosphate-binding arginine residues are in perfect alignment for ion coordination. Consistent with 5-FU not effectively stabilizing this interdomain conformation, the resulting structure is found partially open and the phosphate site unoccupied.

## Discussion

Human uridine phosphorylase has been of interest to clinical researchers for several decades now due to its important role in activating front-line chemotherapeutic fluoropyrimidine nucleosides [1]. It has also been studied as the molecular target for the design of specific inhibitors intended to boost plasma and tissue uridine levels in order to rescue normal tissues from these cytotoxic compounds [10]. The structure of hUPP1 in complex with 5-FU reported here clarifies several aspects regarding the conformational dynamics of this enzyme, an understanding of which impacts the rational design of better inhibitors with improved affinity and selectivity. In contrast with better characterized microbial UPPs, the dimeric architecture of the human enzyme leads to interdomain motions that “close” the protein around substrates and “open” to facilitate product release. This observation suggests a new avenue for the development of a novel class of UPP inhibitors that sterically block the closing of this enzyme. Even a small obstruction that maintains the separation of Arg94 from Arg138 (as produced by crystal contacts in the 5-FU-bound structure) may effectively completely disrupt the enzyme's activity by preventing phosphate coordination and in turn, catalyzed phosphorolysis of uridine. Further, understanding the conformational flexibility within the backside loop proximal to the active site provides new approaches as to how to rationally redesign the hydrophobic modifications of acyclouridine analogues to most effectively maximize favourable interactions with low energy configurations of this part of the enzyme. Given recent reports indicating that BAU may also affect human aldehyde oxidase activity [24], improving this compound's selectivity may be critical to creating a therapeutically valuable medicine with limited side-effects.

The implications of this research are gaining significance as new roles for uridine in the cytoprotection of tissues are being discovered. Recent reports have shown that uridine phosphorylase activity is under the regulation of a number of hepatic nuclear receptors, suggesting a link between lipid and uridine metabolism [25], [26]. This is increasingly of interest given the rising prevalence of fatty liver disease among populations. Uridine has also been found to protect astrocytes from cellular death under energy-limiting conditions, such as ischemia [27], [28]. Further, administration of uridine in combination with docosahexaenoic acid is being tested as a potential treatment for both Alzheimer's disease [29] and Parkinson's disease [30]. Thus, targeting human UPP to raise endogenous uridine levels may prove valuable as a more general approach toward the cytoprotection of a variety of cells, beyond its original application as a means of rescuing tissues exposed to fluoropyrimidines during the course of chemotherapeutic treatment.

## Materials and Methods

### Protein production and purification

Production and isolation of hUPP1 was conducted as previously reported [20], [31] and followed standard laboratory protocols for recombinant bacterial protein expression and purification. In brief, pQE plasmid containing an N-terminally six histidine-tagged construct of the enzyme was transformed into BL21(DE3) *E. coli*. Freshly transformed colonies were cultured in Terrific Broth and induced with 0.1 mM isopropyl-β-D-thiogalactopyranoside (IPTG) at an O.D. of 1.0. Growth was continued overnight at 18°C. Cells were harvested and resuspended in 50 mM Tris buffer pH 8.0, 300 mM KCl, 10% glycerol with 20 mM imidazole. The bacteria were then disrupted by sonication on ice and membranes with other insoluble material were pelleted by high speed centrifugation (100,000×g). Recombinant hUPP1 was subsequently purified from the resulting supernatant using Ni-NTA affinity chromatography and batch eluted with 500 mM imidazole added to the sonication buffer above. Further purification was conducted using gel filtration chromatography over Superdex 200 resin equilibrated in 300 mM KCl, 50 mM Tris buffer pH 8.0 with 1 mM Tris (2-carboxy-ethyl) phosphine (TCEP). The final sample was verified to be homogenous by SDS-PAGE experiments and used directly for crystallization.

### Crystallization

Purified hUPP1 at 4 mgs/mL was supplemented with 1 mM 5-FU (Sigma) and subject to crystallization screening in conditions similar to those previously identified to successfully crystallize hUPP1 with BAU [20]. Large rod-shaped crystals formed in 17% PEG 3350, 100 mM Bis-Tris buffer pH 5.5, and 160 mM MgCl_2_. Crystals were frozen by submersion in liquid nitrogen after a few seconds incubation in cryoprotectant containing the above constituents supplemented with 25% ethylene glycol and 5 mM 5-FU.

### Data collection/processing and structure determination

Data was collected at SSRL beamline 7-1 as summarized in Table 1. A complete, high quality dataset to 2.3 Å resolution was collected. This data was processed and reduced by the HKL2000 package with Denzo and Scalepack [32]. The 5-FU-bound hUPP1 crystallized in the same orthogonal space group (P2_1_2_1_2_1_) as crystals of this protein in complex with BAU and possessed low mosaicity. Molecular replacement phasing of the data obtained on hUPP1 with 5-FU was successful through Molrep [33] using monomers of hUPP1 with BAU as a search model (PDB ID: 3EUF) [20]. Solution phases were sufficient to resolve unambiguous density for the four unmodeled 5-FU ligands (one per protein chain). Rounds of model building and refinement were performed using Coot [34] and Refmac [33]. As with the BAU-bound structure, there is a lack of electron-density for the first 15 residues of hUPP1, the N-terminal cloning artifact residues ‘MRGSHHHHHHGSPGLQEF’, and the final two C-terminal residues. Tight non-crystallographic symmetry restraints (between 4 chains) were retained for the main chain loop residues 78–84 due to the low quality of the electron-density map in this region of the protein. The final structure was refined with Refmac to an R_factor_/R_free_ of 22.5%/27.7%, with approximately 89% of residues in most favourable regions of the Ramachandran plot as analyzed by Procheck [35]. The model was further validated using Molprobity [36], scoring in the 98^th^ percentile. Figures were rendered using ICM Browser-Pro (Molsoft) or LigPlot [37]. The atomic coordinates and structure factors have been deposited in the Protein Data Bank (3NBQ).

**Table 1:** Summary of crystallographic data and model refinement statistics.

Diffraction Data:		
Source	SSRL 7-1	
Λ	0.98 Å	
Space Group	P2_1_2_1_2_1_	
Cell constants	a = 61.32 Å	
	b = 85.28 Å	
	c = 260.58 Å	
Mosaicity	0.20°	
Resolution	50-2.30 Å	(2.38-2.30 Å)
Rmerge	5.9%	(24.7%)
I/σ	19.3	(2.7)
Completeness	94.1%	(67.4%)

Model Refinement:		
Number of reflections	58,444	
Number of monomers/A.U.	4	
Atoms/A.U.	9176	
Protein	9012	
Ligand (Four 5-FU molecules)	36	
Water	128	
Rcryst	22.5%	
Rfree	27.7%	
Rmsd bond lengths	0.011 Å	
Rmsd bond angles	1.30°	
Ramachandran statistics		
Most favored regions	89.4%	
Additional allowed regions	10.6%	
Molprobity Score	98%	
